# Difficulties in Differentiating Osteosclerosis in Patients With Multifocal Micronodular Pneumocyte Hyperplasia and Cancer

**DOI:** 10.7759/cureus.35659

**Published:** 2023-03-01

**Authors:** Makiko Kumamoto, Kaoru Hamada, Chiho Ohbayashi, Shinji Tamaki, Shigeo Muro

**Affiliations:** 1 Department of Respiratory Medicine, National Hospital Organization Nara Medical Center, Nara, JPN; 2 Department of Respiratory Medicine, Nara Medical University, Kashihara, JPN; 3 Department of Diagnostic Pathology, Nara Medical University, Kashihara, JPN

**Keywords:** multiple sclerotic bone lesions, mtor, cisplatin, tuberous sclerosis complex, multifocal micronodular pneumocyte hyperplasia

## Abstract

A 52-year-old woman with multifocal micronodular pneumocyte hyperplasia in bilateral lungs and multiple sclerotic bone lesions (SBLs) visited our hospital. Tuberous sclerosis complex (TSC) was suspected but did not meet the diagnostic criteria. Ten years later, at age 62, the patient developed ureteral cancer. Cisplatin-containing chemotherapy ameliorated ureteral tumor, concomitant with exacerbation of SBLs. It was difficult to distinguish whether the exacerbation of SBLs was due to exacerbation of TSC or bone metastasis of cancer. The administration of cisplatin made the diagnosis even more difficult because its molecular biological effects can exacerbate the complications of TSC.

## Introduction

Multifocal micronodular pneumocyte hyperplasia (MMPH) and sclerosing bone lesions (SBLs) are known complications of tuberous sclerosis complex (TSC). SBLs, in particular, are a frequent complication of TSC but often go unexamined and undetected because most cases are asymptomatic. When a patient with TSC develops cancer, a full-body examination for cancer staging may result in the discovery of SBLs. In such cases, it is necessary to differentiate whether the SBLs are complications of TSC or osteosclerotic bone metastases of cancer to determine a treatment plan. The effectiveness of bone scintigraphy in distinguishing between these two possibilities remains controversial. The diagnostic rate of metastatic bone tumors by bone biopsy is considered high, but it is invasive and can fail to diagnose the disease. In addition, cisplatin is often used for the treatment of cancer, but, theoretically, the molecular biological effects of cisplatin can exacerbate the bone complications of TSC. Thus, cisplatin administration makes the diagnosis of SBLs in cancer patients with TSC more difficult. Here, we report a case in which a patient with MMPH and SBLs and suspected TSC developed cancer, and her SBLs worsened after cisplatin administration, making it difficult to distinguish between worsening SBLs due to TSC and worsening bone metastasis due to cancer.

## Case presentation

**Figure 1:**
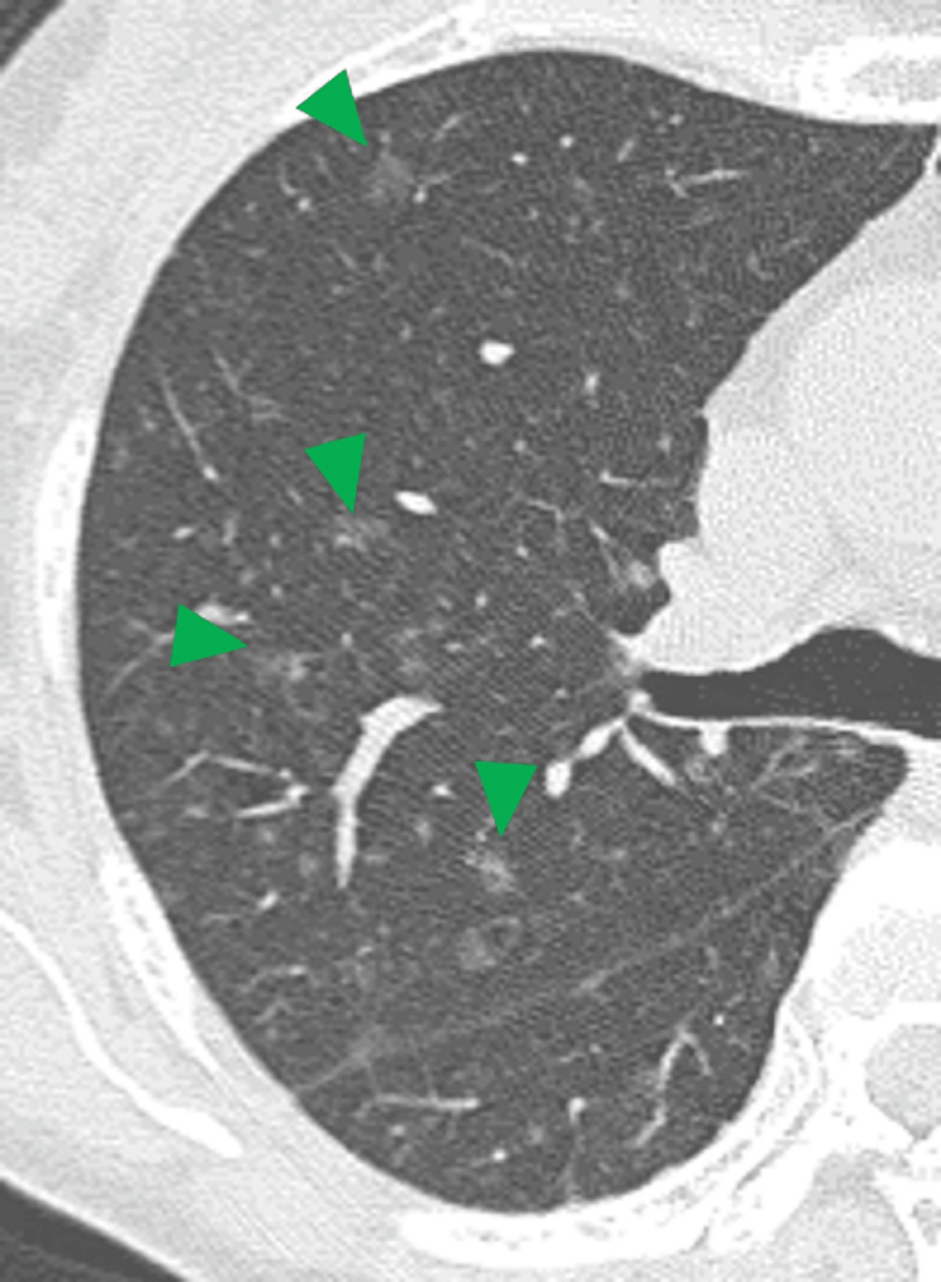
Chest CT scan shows multiple GGOs in both lungs (green arrowheads). GGOs = ground-glass opacities

A 52-year-old woman presented to our hospital with a chief complaint of neck pain. Cervical CT showed no abnormalities in the neck, but abnormal shadows were seen in the upper lung fields that were within the imaging range. Whole-body CT scan was performed, which showed multiple ground-glass opacities (GGOs) ranging in size from a few mm to 1 cm in the bilateral lungs (Figure 1).

**Figure 2:**
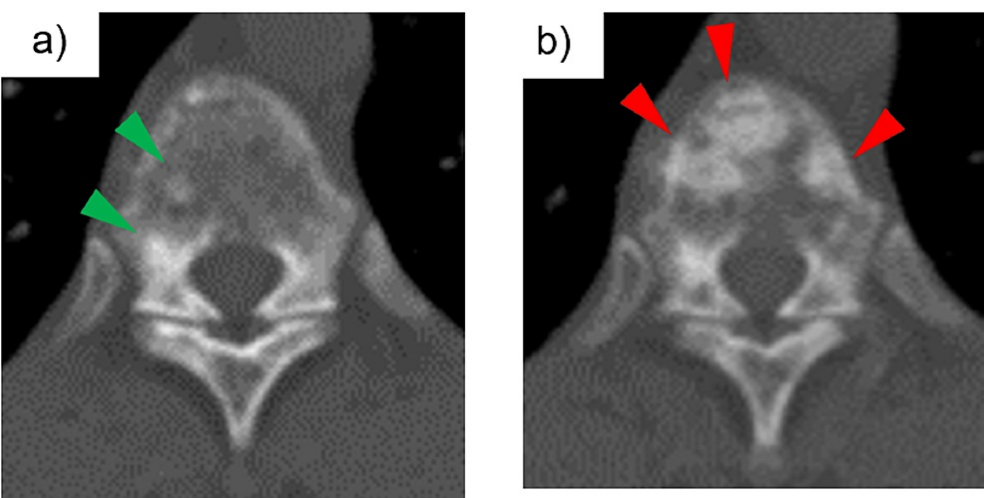
Multiple SBLs seen at the initial visit (green arrowheads) (a). After two courses of cisplatin/gemcitabine chemotherapy, there was a clear exacerbation of SBLs (red arrowheads) (b). SBLs = sclerosing bone lesions

CT also showed multiple SBLs (Figure 2, Panel a).

However, there were no respiratory symptoms, such as cough or shortness of breath, bone pain, or fever. At the time of presentation, her vital signs included a normal body temperature, blood pressure of 120/78 mmHg, pulse rate of 84 beats/minute, respiratory rate of 12 breaths/minute, and oxygen saturation (SpO_2_) of 97% on room air. On her physical examination, there was no cervical or supraclavicular adenopathy. Lung examination revealed clear and equal breath sounds bilaterally, and no wheezing, crackles, or rhonchi. Biochemical tests and tumor markers for lung cancer were found to be within the normal ranges.

**Figure 3:**
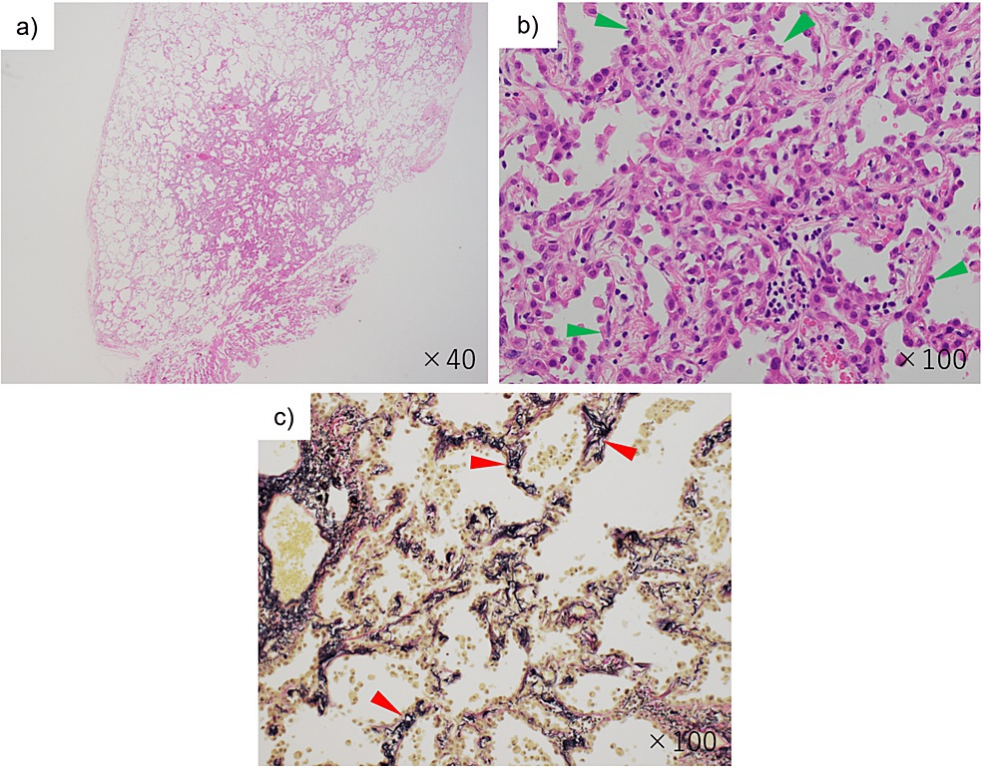
The pathological findings of lung GGOs. H&E staining image shows a single-layered hyperplasia of type II pneumocyte-like cells without atypism (green arrowheads) (a: low-magnification, b: high-magnification). EVG staining image shows fibrous thickening of the alveolar walls (red arrowheads) (c). H&E = hematoxylin and eosin; EGV = Elastica van Gieson

Video-assisted thoracoscopic lung biopsy confirmed the diagnosis of GGO as MMPH (Figure 3).

**Figure 4:**
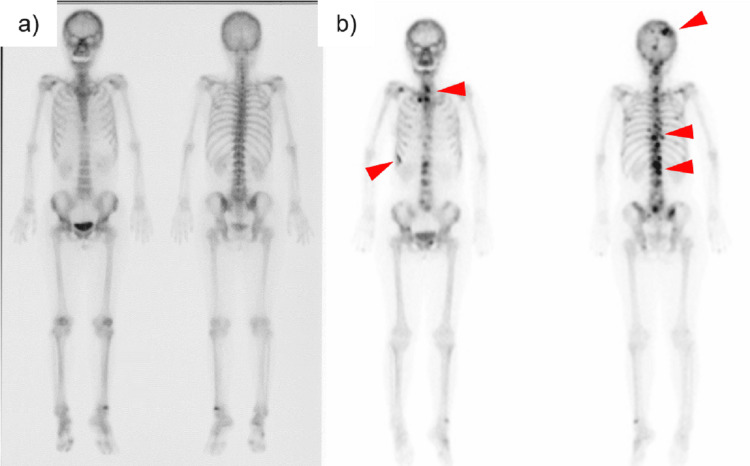
Bone scintigraphy showing no abnormalities in bone metabolism at the time of the initial diagnosis (a). After two courses of chemotherapy, it shows increased uptake in vertebrae, ribs, pelvic bone, and cranial bone (red arrowheads) (b).

Bone scintigraphy was performed for a close examination of osteosclerosis which showed a negative appearance (Figure 4, Panel a).

TSC was suspected due to the coexistence of MMPH and SBLs, which are known complications of TSC. However, there were no abnormal findings on her brain MRI, no epileptic episodes, or TSC-specific skin lesions. She did not meet the diagnostic criteria for TSC because she did not want genetic testing and had no abnormal findings or family history of TSC other than MMPH or SBLs. MMPH and SBLs were followed up and both were stable for 10 years.

When she turned 62 years old, she visited urology because of gross hematuria. Abdominal CT showed wall thickening of the right ureteropelvic junction and intra-abdominal lymphadenopathy. A ureteroscopic biopsy diagnosed right ureteral cancer. Because there were no obvious distant metastases, a nephroureterectomy was scheduled after preoperative chemotherapy. After two courses of cisplatin and gemcitabine chemotherapy over two months, the wall thickness at the right ureteropelvic junction and intra-abdominal lymphadenopathy improved. At this time, SLBs were clearly exacerbated (Figure 2, Panel b). In contrast, pulmonary GGOs were unchanged. The cause of the exacerbation of SBLs was considered to be either exacerbation of bone lesions of TSC or osteoblastic bone metastases of ureter cancer. To ensure the diagnosis of the new bone lesions, bone scintigraphy and bone biopsy of the first lumbar vertebra were performed. The scintigraphic pattern showed increased uptake in vertebrae, ribs, pelvic bones, and skull (Figure 4, Panel b), but no malignant findings were detected on bone biopsy of the first lumbar vertebra. The new bone lesions were determined not to be metastases, and an open nephroureterectomy was performed as planned. Surgery revealed para-aortic lymph node metastases, and adjuvant chemotherapy was planned after the patient’s general condition improved. However, one month after surgery, CT revealed further deterioration of SBLs, and laboratory findings revealed pancytopenia. A bone marrow aspiration was performed from the iliac bone to investigate the cause of pancytopenia, which showed foci of polygonal cells with enlarged round nuclei and pale acidic cytoplasm. Although the cell morphology was not identical to that of ureteral carcinoma cells, immunostaining showed GATA3(+), CD10(-), CK7(+), CK20(+), and PAX8(-), suggesting metastasis from ureteral carcinoma. Therefore, the cause of the spread of bone lesions was diagnosed as bone metastases of ureteral cancer. Her condition subsequently worsened, and she died one year after the onset of ureteral cancer. No autopsy was performed.

## Discussion

TSC is an autosomal dominant disease, characterized by hamartoma formation in various organs. The disease incidence is one out of 6,000 people, and two-thirds of the patients are sporadic cases [1,2]. It results from mutations in one of two genes, *TSC1 *(encoding hamartin) or *TSC2 *(encoding tuberin). Pulmonary manifestations of TSC are lymphangioleiomyomatosis and MMPH. Although the frequency of MMPH due to TSC is generally considered to be less than 1%, it has been reported to occur as frequently as 32.3% in autopsies of patients with TSC [3]. In our case, neither clinical nor genetic diagnostic criteria for TSC were met because the patient refused genetic examination [4]. Moreover, there was no relevant family history. However, given that MMPH patients have been reported who do not meet the diagnostic criteria for TSC [5] and that SBL is a common symptom in TSC, we speculated that genetic abnormalities in TSC might be involved in some way in our case.

Although SBLs and bone cysts are frequent manifestations of TSC, especially SBL, which is reported to be present in 98% of TSC patients [6], they are not included in the diagnostic criteria for TSC. SBL associated with TSC is usually asymptomatic and does not require treatment. However, in patients with TSC with malignancy, it is important to distinguish whether the bone lesions are SBLs associated with TSC or osteosclerotic metastases. SBLs associated with TSC have been reported to show a negative appearance on bone scintigraphy in some case reports [7,8]. Therefore, bone scintigraphy is useful in differentiating bone lesions. On the other hand, Jonard et al. reported SBLs in patients with TSC show a positive appearance on bone scintigraphy [9]. As a possible explanation for this discrepancy, Song et al. suggested that bone scintigraphic uptake of TSC-derived SBL is affected by patient age (25 years for positive cases and 64 and 72 years for negative cases), i.e., the metabolic activity of SBL may be higher in younger than in older patients [7]. Although there are no reports about the natural course of bone lesions in adult TSC patients, there is a report in the pediatric literature supporting this idea. Boronat et al. reported that of 50 pediatric patients whose bones were examined by MRI, 19 showed a new appearance of osteosclerosis and 14 showed lesion enlargement [10]. These data may suggest that bone lesions may be more active in younger TSC patients. Because each clinical manifestation of TSC appears at a specific time, childhood may be the time when osteosclerosis of TSC appears.

Animal studies suggest that the *TSC1 *gene is involved in the development of osteosclerosis in TSC. The malfunction of the *TSC* gene activates the rapamycin complex 1 (mTORC1) signaling, inducing the appearance of bone sclerosis on images and osteogenesis imperfecta [11-13]. On the other hand, cisplatin has been reported to activate the Akt/mTOR survival pathway [14]. In our case, while the ureteral tumor improved with chemotherapy including cisplatin, there was an exacerbation of SBL. Because a bone biopsy of the first lumbar vertebra did not reveal any malignancy, we considered the possibility that the exacerbation of the originally present TSC due to activation of the Akt/mTOR survival pathway by cisplatin was the cause of the SBL exacerbation. As far as we know, there are no reports discussing the direct effect of cisplatin on TSC patients. On the other hand, there is a report that the effect of cisplatin on TSC mutation-positive lung cancer depends on the variation of the *TSC *mutation [15]. It suggests the possibility that cisplatin may affect TSC lesions depending on the variation of *TSC *gene mutation.

In our case, bone scintigraphy showed Tc-99m HMDP uptake in the bone lesions, but as mentioned above, active SBL of TSC can be positive on bone scintigraphy, and bone metastases cannot be distinguished from bone lesions of TSC. Bone biopsy of the first lumbar vertebrae did not diagnose bone metastases, possibly because the lesion site was not sampled. Eventually, pancytopenia appeared, and bone marrow aspiration from the iliac bone revealed that the spread of SBL was due to bone metastasis of ureteral cancer. The question arises, then, why did the bone metastases worsen when the primary site of ureteral cancer improved with chemotherapy? We speculated that differences in the microenvironment and the degree of genetic abnormalities between the primary site of the tumor and metastatic bone lesions may have influenced the clinical course.

The present case shows the difficulty in differentiating the etiology of osteosclerosis exacerbation in cancer-bearing TSC patients. This difficulty arose because (1) despite successful shrinkage of the primary site of the tumor by administration of anticancer drugs, exacerbation of osteosclerosis was observed, and (2) cisplatin administration may have exacerbated osteosclerosis associated with TSC by activating mTORC1.

## Conclusions

We present a case of a cancer patient with suspected TSC who presented with MMPH and osteosclerosis. It was difficult to distinguish whether the exacerbation of osteosclerosis seen after chemotherapy was due to the progression of tuberous sclerosis syndrome or bone metastases of cancer; the administration of cisplatin to a patient with TSC may make the distinction even more difficult. Bone scintigraphy is not useful for differentiation, and we believe that repeat bone biopsy is the only way to differentiate.
